# Editorial: Biology of non-canonical nucleic acids from humans to pathogens

**DOI:** 10.3389/fmicb.2022.981679

**Published:** 2022-07-18

**Authors:** Ilaria Frasson

**Affiliations:** Department of Molecular Medicine, University of Padua, Padua, Italy

**Keywords:** non-canonical, DNA and RNA, G-quadruplex (G4), pathogens, antimicrobial

Researchers have spent the last decades trying to solve the mysteries of how nucleic acids regulate a considerable variety of biological processes. We have gained considerable knowledge from the results of the Human Genome Project, as well as, from all the fully sequenced genomes of microorganisms present in the scientific databases. Indeed, it is now clear that nucleic acids regulate key biological processes, such as transcription, replication, gene expression, and others, not just *via* primary nucleotide sequence but also through dynamic tridimensional structures that DNA and RNA sequences can adopt (Varshney et al., 2020). Nucleic acid structures that differ from the established double helix with its covalent bonds are referred to as “non-canonical” nucleic acid structures. Among the non-canonical structures, hairpins, cruciform, R-loops, G-quadruplex (G4), and i-motifs (iM) have been extensively characterized *in vitro* (Takahashi and Sugimoto, 2020). Their folding/unfolding has been challenged also in cellulo (Lightfoot et al., 2019). Recently, G4s, which can form in regions enriched in guanine residues, stood out for their structural polymorphism coupled to complex functional roles at the human cellular level (Matsumoto and Sugimoto, 2021). Notably, G4s have been reported to be embedded in the genome of crucial human pathogens, playing crucial biological roles (Perrone et al., 2013a,b; Dumetz and Merrick, 2019; Frasson et al., 2019, 2021; Saranathan and Vivekanandan, 2019; Ruggiero and Richter, 2020; Yadav et al., 2021). G4s have been demostrated to be feasible antimicrobial targets (Ruggiero and Richter, 2018; Ruggiero et al., 2021).

The recent research that focused on G4s and microorganisms worked to set up or implement specific tools to predict their presence in the genome of pathogens (Lavezzo et al., 2018; Cagirici et al., 2022; Vannutelli et al., 2022), as G-rich regions cannot be straightforwardly coupled to the possible folding of G4s. Moreover, the pandemic years we have been through have brought to light the importance of promptly understanding if G4s are embedded in the pathogen's or influence the host-pathogen interaction network, or if they represent feasible antiviral targets. With this aim, Bartas et al. presented the bioinformatics analysis of the G4 landscape in the genome of all sequenced members of the Nidovirales family. Their analysis highlighted the presence of conserved putative G4s in regions categorized as inverted repeats and in 5- or 3′-UTRs, thus regions that correlate with viral RNA packaging, transcription, and translation. Moreover, the authors reported possible G4 binding motifs in human proteins that may bind to the SARS-CoV-2 RNA. Surprisingly, taking a closer look at the genome of human viral pathogens, Ruggiero et al., reported the presence of conserved regions composed of multiple adenine (A), thymine (T), or uracil (U) tracts in principle able to form quadruplex structures. Their analysis proved that As, Ts, and Us do not fold into a multilayered quadruplex structure similar to the G-quadruplex structure, nonetheless, these tracts are prone to fold into a tridimensional structure able to induce polymerase stalling. As, A-tracts have already been reported to occur and to have biological relevance in both eukaryotic and bacteria, Ruggiero et al. suggest that viruses, as well, may exploit the A-tracts to regulate nucleic acids biology (i.e., nucleosome organization, transcription regulation, internal looping, protein recognition, etc.).

Closing up on prokaryotes, Cashchina et al. described the analysis of the genome of bacteria belonging to the Archeae genera, to identify their presence and to observe their evolution-driven conservation. They determined that the majority of the bacteria included in the Methanomicrobiaceae family contained multiple regions possibly forming G4s. A deeper analysis showed that putative G4 forming sequences were followed by a cytosine (C)-rich tract. The presence of these C-tracts induced the formation of alternative structures such as hairpin and antiparallel triplex, that were in equilibrium with the G4 structure in solution, revealing alternative non-canonical structures in the members of Archaea. Besides, Shankar et al. dealt with the persistent concern of the bacterium *Klebsiella pneumoniae*, which causes life-threatening infections in humans. Their analysis aimed at the identification of evolutionary conserved G-quadruplex forming motifs as possible antibacterial targets. Shankar et al. identified six highly conserved G4s in the promoter region of five essential genes, involved in nutrient transport and bacterial metabolism. The administration of the established G4-ligand Braco-19 to *Klebsiella pneumoniae* isolates tackled bacterial growth, paving the way to innovative therapeutic opportunities to fight Klebsiella infections.

G4s and non-canonical nucleic acid structures have emerged as critical regulators of biological processes and potential therapeutic targets against various human pathogens, including viruses, bacteria, and protozoans. We have recently realized how human pathogens can change the world equilibrium and keep humankind in check. Nucleic acid secondary structures possibly finely regulate the infective lifecycles of crucial pathogens and, thus, represent innovative antimicrobial targets to broaden the treatment options for infectious diseases and overcome insidious resistance traits to current antimicrobials.

## Author contributions

The author confirms being the sole contributor of this work and has approved it for publication.

## Conflict of interest

The author declares that the research was conducted in the absence of any commercial or financial relationships that could be construed as a potential conflict of interest.

## Publisher's note

All claims expressed in this article are solely those of the authors and do not necessarily represent those of their affiliated organizations, or those of the publisher, the editors and the reviewers. Any product that may be evaluated in this article, or claim that may be made by its manufacturer, is not guaranteed or endorsed by the publisher.
